# PReS-FINAL-2168: Comparison of safety and retention rate of TNF antagonist therapy in juvenile-onset and adult-onset ankylosing spondylitis: data from the spanish registry biobadaser 2.0

**DOI:** 10.1186/1546-0096-11-S2-P180

**Published:** 2013-12-05

**Authors:** WA Sifuentes Giraldo, C Guillén Astete, ML Gámir Gámir, S Pérez Vicente, L Carmona, JJ Gómez-Reino

**Affiliations:** 1 Rheumatology, University Hospital Ramon y Cajal, Madrid, Spain; 2 Unidad de Investigación, Sociedad Española de Reumatología, Madrid, Spain

## Introduction

Ankylosing spondylitis (AS) is a chronic inflammatory disease that belongs to the group of spondyloarthritis, which involves the spine, peripheral joints and entheses. Juvenile-onset AS affects children under the age of 16 years and present a clinical course different from adult-onset AS. Several randomized clinical trials have shown that TNF antagonists are an effective alternative treatment in adult-onset AS. Similar results have been reported in juvenile-onset AS, but have been fewer studies conducted in this group of patients.

## Objectives

To compare the safety and retention rate of TNF antagonist therapy in patients with juvenile-onset and adult-onset AS.

## Methods

Analysis of patients with adult-onset and juvenile-onset AS included in the Spanish registry BIOBADASER 2.0 (October 2006 to November 2012). Incidence rates (irs) of adverse events (aes) and rates of discontinuation were compared between both groups.

## Results

A total of 686 patients (524 males, 162 females) were included, comprising 33 juvenile-onset AS and 653 adult-onset AS patients. The ages of diagnosis were 11.9 ± 0.7 years and 34.4 ± 0.5 for juvenile-onset and adult-onset AS, respectively. The duration of disease was higher in the juvenile-onset group (17.9 ± 1.9 years) than in the adult-onset group (9.3 ± 0.4) and HLA-B27 positivity was similar in both groups (82.4% and 86.4%, respectively). Axial involvement was higher in adult-onset patients (74.9% vs 63.6%) and peripheral involvement was more common in juvenile-onset AS (45.5% vs 32.5%). The TNF antagonist more frequently used as first treatment was infliximab in both adult-onset (48.5%) and juvenile-onset AS (50%), and sulfasalazine or other DMARD were used concomitantly in 43% and 35.5%, respectively. The irs of aes was largest in adult-onset AS (140.5 events/1000 patient-years, CI 95%: 13.2-15.8) and lowest in juvenile-onset AS (30 events/1000 patient-years, CI 95%: 0.0-1.9), but severe adverse events were similar in both groups (43 events/1000 patient-years, in adult-onset AS [CI 95%: 3.7-5.1], and 44 events/1000 patient-years in juvenile-onset AS [CI 95%: 2.3-7.5]). The rates of discontinuation due to aes and inefficacy were both higher in adult-onset AS (3,7 [CI 95%: 3.1-4.3] and 2,1 [CI 95%: 1.7-2.6], respectively) compared with juvenile-onset AS (2,7 [CI 95%:1,2-5,3] and 1,3 [CI 95%:0,4-3,4], respectively).

## Conclusion

The safety and retention patterns of TNF antagonist therapy were similar in adult-onset and juvenile-onset AS, but discontinuation due to AE and inefficacy were higher in adult-onset group.

## Disclosure of interest

None declared.

